# Draft Genome Sequence of *Nonomuraea* sp. Strain C10, a Producer of Brartemicin, Isolated from a Mud Dauber Wasp Nest in Nepal

**DOI:** 10.1128/MRA.01109-19

**Published:** 2019-11-07

**Authors:** Niraj Aryal, Saefuddin Aziz, Prajwal Rajbhandari, Harald Gross

**Affiliations:** a Department of Pharmaceutical Biology, Pharmaceutical Institute, University of Tübingen, Tübingen, Germany; b Microbiology Department, Biology Faculty, Jenderal Soedirman University, Purwokerto, Indonesia; c Department of Industrial Microbiology, Research Institute for Bioscience and Biotechnology (RIBB), Kathmandu, Nepal; d German Centre for Infection Research (DZIF), Partner Site Tübingen, Tübingen, Germany; University of Maryland School of Medicine

## Abstract

Nonomuraea sp. strain C10 produces the cytotoxic natural product brartemicin. Here, we report its draft genome sequence to get insight into brartemicin biosynthesis and to enable genome mining for novel secondary metabolites.

## ANNOUNCEMENT

As in other countries (1, 2), in Nepal, the mud dauber wasp (Screliphron sp.) nest is used in folk medicine. In order to investigate if microbes are involved in its activity, we isolated and characterized the nest-associated Actinobacteria. In chemical analyses, it became apparent that strain C10 produces the cytotoxic trehalose-derived compound brartemicin (3–7). Based on 16S rRNA gene sequence (1,367 bp) similarity, bacterium C10 was identified as a *Nonomuraea* species. The strains most closely related to C10 are *Nonomuraea* sp. strain 7K523 (GenBank accession number MG770787) and Nonomuraea harbinensis Gsoil-1046 (KY078837), both with 99% sequence identity.

In order to investigate the complete biosynthetic capacity for secondary metabolism and to locate and analyze the biosynthetic gene cluster responsible for brartemicin synthesis, sequencing of this strain was initiated.

Mud dauber wasp nests were obtained from wooden houses in the Bardia district of the southwestern region of Nepal. Nest soil (1 g) was crushed into finer particles and dried for 30 min at 100°C. The resultant dried soil particles were sprinkled over humic acid (8) agar plates and incubated at 30°C. After 2 weeks, strain C10 was isolated, along with 3 other actinobacterial isolates, and repeatedly subcultured and purified.

For genomic DNA (gDNA) isolation, strain C10 was then grown in seed medium, which consisted of soluble starch (1%), glucose (0.5%), N-Z-Case (0.3%), yeast extract (0.2%), tryptone (0.5%), K_2_HPO_4_ (0.1%), and MgSO_4_ (0.05%) (pH 7.0), for 8 days at 30°C on a rotary shaker (160 rpm). Subsequently, the Qiagen genomic DNA purification kit was used in combination with 100/G Genomic-tips according to the manufacturer’s protocol, except that for the bacterial lysis the handled volumes were doubled, and incubation time at 50°C was prolonged until a clear lysate was obtained.

Next-generation sequencing was performed at 1,075× coverage using a PacBio Sequel platform with a 10-kb singleplex genomic library and 1 Sequel single-molecule real-time (SMRT) cell, obtaining 1,421,185 reads with an average length of 8,152 bp. No quality filtering was conducted; however, subreads shorter than 50 bp were discarded. The remaining PacBio reads were assembled using SMRTLink v6 and the Hierarchical Genome Assembly Process v4.0 (HGAP4.0) with default parameters and an expected 13-Mbp genome size that was based on previously determined *Nonomuraea* genome sizes (9–11). The draft genome of *Nonomuraea* sp. strain C10 consists of 3 scaffolds (*N*_50_, 6,800,706 bp; *L*_50_, 3), a total of 9,416,283 bp, and a G+C content of 71.4%. Gene functional annotation using the NCBI Prokaryotic Genome Annotation Pipeline (PGAP v4.8) (12) identified 8,473 coding genes.

Automated secondary metabolism analysis using AntiSMASH 5.0.0 (13) predicted 21 biosynthetic gene clusters.

### Data availability.

This whole-genome shotgun project and the partial 16S rRNA gene sequence have been deposited at DDBJ/ENA/GenBank under the accession numbers VPFF00000000 and MN400757, respectively. The raw sequencing data are available from the Sequence Read Archive (SRA) under the accession number SRR9964087.
